# Beyond the first purchase: unpacking the continuance intentions behind sustainable consumption

**DOI:** 10.3389/fpsyg.2025.1627728

**Published:** 2025-10-10

**Authors:** Xiayu Tong, Kai Wang, Shuaiming Feng

**Affiliations:** ^1^School of Education Science, Nanjing Normal University, Nanjing, China; ^2^College of Entrepreneurship, Zhejiang University of Finance and Economics, Hangzhou, China; ^3^School of Management, Zhejiang University of Finance and Economics, Hangzhou, China; ^4^Yingyang School of Financial Technology, Zhejiang University of Finance and Economics, Hangzhou, China

**Keywords:** continuance behavior intention, theory of planned behavior, pro-environmental self-identity, pre-adoption experience, habit

## Abstract

This study investigates the continuance behavior intention (CBI) of Chinese consumers toward green products by extending the Theory of Planned Behavior (TPB) with pre-adoption experience (PAE), environmental perception (EP), attitude (ATT), subjective norms (SN), perceived behavioral control (PBC), habit (HAB), and pro-environmental self-identity (PESI). Data from 588 respondents with prior green product experiences were analyzed using partial least squares structural equation modeling (PLS-SEM). Results show that PAE positively influences ATT, SN, and PBC. EP significantly affects ATT, SN, and PBC, while HAB is crucial for sustaining green consumption. Additionally, PESI moderates the relationships among EP, ATT, HAB, and CBI. This study highlights the importance of aligning green products with consumer identities and promoting PESI through education and incentives, which provides practical insights for fostering sustained green consumption in emerging markets.

## 1 Introduction

The growing awareness of environmental issues and the recognition of ecological degradation’s severity have underscored the importance of sustainable consumption in society (Hasan et al., 2024). The prevalent consumption habits (HABs), which rely heavily on finite resources and fluctuating energy markets, are detrimental to environmental and societal sustainability. As a solution to the challenges posed by these traditional HABs, sustainable consumption behavior (SCB) has come to the forefront (Wang et al., 2025). The concept of SCB has arisen in response to pressing environmental concerns (Lavuri et al., 2023) and is characterized by consumption practices that satisfy ecological demands (Ghaffar and Islam, 2024), safeguard the environment (Ali et al., 2023), and ensure the longevity of societal well-being (Hasan et al., 2024). The United Nations has outlined sustainable development goals, which include strategies for promoting sustainable consumption (Poonamallee, 2025), urging us to boost resource efficiency, endorse sustainable living, and transition towards a green, sustainable economy. Consequently, the movement towards eco-friendly lifestyles is gaining momentum, and it is crucial for all involved parties to endorse this shift (Tavitiyaman et al., 2024). Embracing SCB can mitigate adverse environmental effects and foster better living conditions (Wang et al., 2025). SCB is gaining societal acceptance due to its potential for long-term benefits and its capacity to enhance societal living standards (Wang et al., 2024).

Previous studies have addressed the motivation-adoption link (i.e., how to motivate sustainable consumer behaviors) (Yang et al., 2025) rather than the adoption-continuance link (i.e., how to maintain those behaviors) (Gupta et al., 2020). This gap reflects a fundamental debate in environmental psychology that whether behavioral persistence is driven primarily by conscious intention (Ajzen, 1991) or requires additional mechanisms like automated HABs (Verplanken and Aarts, 1999), identity reinforcement (Whitmarsh and O’Neill, 2010), and experiential learning (Agarwal et al., 2025). Gupta et al. (2020) stated that more empirical evidence should be explored to deepen the understanding of the determinants that influencing consumers’ adoption-continuance behavior. Therefore, this study focuses on the continuance intention, through a perspective of Chinese consumers who have the post-adoption experience in purchasing the green product based on a quantitative research method to further explore the continuance of Chinese consumers in sustainable consumption behavior intention.

Numerous scholars have delved into the various factors influencing sustainable consumption behavior, such as demographic characteristics, personal values, ecological awareness, and its correlation with corporate culture (Ogiemwonyi, 2024; Wu and Long, 2024). The individual’s attitude (ATT) plays a pivotal role in shaping environmental actions and is a key factor in the persistence of sustainable consumption practices (Gansser and Reich, 2023). Despite a wealth of research on sustainability and consumer actions, there remains a gap in understanding the transition from green consumption to the ongoing engagement in sustainable consumer behavior. For example, while research indicates that the reasons for maintaining behavior are often framed within the same theoretical frameworks as those for initiating behavior, the specifics, orientation, and significance of these frameworks can vary significantly from the start to the ongoing phase (Zheng et al., 2023). The perceived effectiveness of a product might drive the initial purchase, but its influence wanes with continued use and sustainable consumption (Granato et al., 2022). Therefore, future research should concentrate on the post-adoption phase of the sustainable consumer journey. Additionally, many experts argue that the literature is lacking a comprehensive model for long-term sustainable consumer behavior, despite the existence of various models (Agarwal et al., 2025). This study is thus prompted by the question, “What are the key factors that influence the continuation of consumers’ engagement in sustainable consumption behavior?”

Beyond other theoretical frameworks, Theory of Planned Behavior (TPB) has been widely employed by researchers to comprehend environmental actions (De Leeuw et al., 2015). TPB posits ATT, subjective norms (SN) and perceived behavioral control (PBC) are interconnected with behavior. The academic literature indicates that integrating TPB with additional constructs can deepen our comprehension of the continuity of sustainable consumer behavior (Gansser and Reich, 2023). For instance, research expanding on TPB has demonstrated that various personal behavioral and cognitive factors, such as moral attitudes, health consciousness, and perceived external environmental impacts, significantly contribute to sustaining green consumption behavior (Qi and Ploeger, 2021).

Additionally, most prior studies have focused on economic influences such as products’ quality (Yan et al., 2024), consumers’ emotions (Taufique, 2020), and psychological influences (Joshi and Rahman, 2019). Whereas, experience is proven as a vital variable influencing consumers’ re-purchase intention (Li, 2025), scarce literature has investigated how past-purchasing experience affected the consumers’ adoption-continuance behavior toward green products. What’s more, self-identity was suggested as a significant explaining variable when discussing the consumer behavior (Zheng et al., 2023). Within the sustainable research field, pro-environmental self-identity (PESI) can be characterized as an individual’s perception of themselves as proponents of environmental actions (Lavuri et al., 2023). People select actions that align with or enhance their self-conception, often driven by a desire for self-expression in their consumption choices (Paquin and Keating, 2017). As noted by Lage et al. (2022), self-awareness can vary among individuals based on their unique experiences and cultural contexts. It is therefore crucial to investigate the potential variations in how individuals perceive the adoption and continuation of green product consumption in relation to their PESI (Dermody et al., 2015). Under consistent decision-making conditions, individuals can develop patterns of sustainable consumer behavior over time, which eventually become habitual as an automatic response (Ghaffar and Islam, 2024). While HABs have been extensively studied in relation to past behavior and behavior maintenance, their integration with SCB in the context of pre-adoption experience and post-adoption has been less explored.

To address the growing need in the literature for a more comprehensive understanding of the factors influencing SCB, particularly in the post-adoption phase, this study integrates pre-adoption experience (PAE), environmental perception (EP), habit (HAB), pro-environmental self-identity (PESI), and continuance behavior intentions (CBI) toward green products into the Theory of Planned Behavior (TPB) framework as a new holistic model on consumers’ continuance sustainable behavior intention.

To this end, the article can contribute to a better understanding of the importance of various factors influencing SCB, taking into account the increasingly deteriorating environment and growing ecological ATTs of consumers (Saltik and Akova, 2024). First, this study proposes a new holistic model that comprehensively delineates the facets of the SCB continuance phase and broadens its scope to forecast the persistence of sustainable consumer actions. Second, the study aims to explore both the direct and indirect effects of EP and PAE on CBI. The study’s novelty also lies in employing the construct of PESI to elucidate the ongoing commitment of consumers to sustainable consumption. While previous investigations have underscored the role of self-identity in the purchase intentions for eco-friendly products, the discussion on how this influences the continuation of sustainable consumption behaviors has been largely overlooked (Becerra et al., 2023). This study aims to fill this gap by examining how PESI influences the continuation of sustainable consumption behaviors. Additionally, HABs have predominantly been viewed as a straightforward predictor of purchase intentions (Tavitiyaman et al., 2024), with their influence on CBI beyond the first purchase being seldom examined. This study explores the role of HABs in sustaining green consumption behaviors over time.

## 2 Literature review and theoretical framework

### 2.1 Theory of planned behavior and continuance intention

Theory of planned behavior, initially proposed by Ajzen (1985, 1991), builds upon the Theory of Reasoned Action (TRA) (Ajzen and Fishbein, 1980) by adding PBC to improve prediction accuracy. TPB posits that ATT, SN, and PBC collectively influence the formation of behavioral intention and subsequent actions (Ajzen, 1985, 1991). TPB has been widely applied across various fields, including green marketing (Rahman and Reynolds, 2019). As detailed below, the fundamental concepts of the TPB are discussed.

Attitude is the individual’s overall appraisal of performing a behavior, stemming from behavioral beliefs and outcome evaluations (Ajzen and Fishbein, 1980). Behavioral beliefs are the individual’s convictions about the outcomes of a specific behavior, while outcome evaluations are their judgments on the desirability of those outcomes (Ajzen, 1991). SN measures the social influence on an individual to perform a behavior, considered a social factor (Ajzen and Fishbein, 1980). SN result from the interplay of normative beliefs and the motivation to comply with them. Normative beliefs involve an individual’s perception of what important others expect in a given context, whereas the motivation to comply indicates the individual’s readiness to follow the expectations of these significant others (Ajzen, 1991). PBC reflects the individual’s perception of the ease or difficulty in performing a behavior (Ajzen and Fishbein, 1980). It is designed to capture perceived/actual resources and opportunities, akin to self-efficacy. It serves as a control mechanism for the adoption or rejection of ATTs and behaviors (Rodgers and Brawley, 1996). PBC is derived from an individual’s control beliefs and their perceived effectiveness. Control beliefs are the individual’s beliefs about factors that may facilitate or hinder the performance of a specific behavior (e.g., time, money, and opportunity). Perceived effectiveness is the individual’s assessment of how influential these factors are in promoting or impeding the behavior in question (Ajzen, 1991).

Theory of planned behavior posits that the immediate precursor to volitional behavior is the intention to perform that behavior. Intentions are viewed as the motivational factors that drive a specific action (Ajzen, 1991). ATT, SN, and PBC are believed to influence behavior indirectly through intentions (Armitage and Conner, 2001). Behavioral intention signifies an individual’s preparedness to embrace a certain behavior, presumed to be the immediate antecedent to action (Ajzen, 2002). The more favorable the ATT towards the behavior, the more supportive the SN, and the greater the PBC, the stronger the individual’s intention to engage in the behavior becomes.

Economic factors play a crucial role in shaping sustainable consumption behavior. Perceived value, affordability, and the economic benefits of green products significantly influence consumers’ intentions to adopt and continue using such products. For instance, studies have shown that consumers are more likely to engage in sustainable behaviors when they perceive a high value for money and when green products are competitively priced (Ghaffar and Islam, 2024). This economic dimension is particularly relevant in the context of PAE and PBC, as these constructs are directly influenced by the perceived economic feasibility of sustainable choices.

### 2.2 Pre-adoption experience

Adoption denotes the initial instance when an individual translates their intentions into action and partakes in a specific sustainable behavior (Ajzen, 1991). Research has indicated that prior experiences can significantly distinguish the behavioral intentions between pre-adopters and post-adopters (Karahanna et al., 1999).

There is a dearth of studies examining consumer PAE as a harbinger of subsequent sustainable purchasing behavior (Fishbein and Ajzen, 1975). The gap in understanding the continuity of PAE behavior, coupled with a lack of clarity on this matter and scant research on sustainable purchasing, prompted the authors to pinpoint crucial factors influencing consumer sustainable behavior (Ajzen, 2002). Furthermore, environmental degradation (including ecological contamination and natural resource depletion) and the consequent heightened focus by firms on manufacturing sustainable products provide additional impetus for this research (Nicolae, 2024). In line with the self-perception theory (Bem, 1967), individual actions can shape one’s ATTs, emotions, and other behavioral factors, suggesting that PAE is specifically selected to address the “experience-intention paradox” in environmental persistence (Fazio, 1987). While initial green purchases often decrease due to cost or performance concerns (Ketelsen et al., 2020), positive PAE creates cognitive scripts that bypass deliberation in continuance decisions (Zacher et al., 2023), which resolves TPB’s oversight of how procedural knowledge sustains behavior when motivation fluctuates. This study defined PAE with a focus on economic satisfaction (e.g., with price discounts), a salient dimension of the initial trial experience for price-sensitive consumers (Si et al., 2022; Chang et al., 2024). For Chinese consumers in particular, positive experiences with economic benefits, such as perceived value from discounts, serve as a critical trigger for initial trials and can form a foundational positive perception that influences subsequent attitudes (Li and Kim, 2024).

### 2.3 Environmental perception

Environmental perception is a multifaceted concept that extends beyond traditional perceptions, encompassing perceptual, cognitive, imaginal, affective, and value aspects (Saarinen, 1976). This concept has been explored through various methodologies and techniques, highlighting its complexity and richness.

Recent advancements in environmental psychology have emphasized the importance of integrating various cognitive and affective processes to understand how individuals perceive and interact with their environment (Joshi and Rahman, 2019). This shift moves away from compartmentalizing mental functions into distinct, isolated processes and toward understanding the individual as a complex, integrated system (Wang et al., 2025).

In the context of maintaining sustainable consumer behaviors, perceived green value has been a frequent area of study, especially in the organic food domain and green restaurants. Research has shown that the perceived quality of green products, which reflects their environmental value, has a positive impact on the formation of continuance intentions and loyalty (Gardner and Abraham, 2010). On the contrary, the perceived risk associated with green products can inversely influence post-purchase intentions to engage in green consumption (Chen and Chang, 2012). ATTs serve as a direct predictor of the likelihood to revisit (Hogg et al., 2024) and an indirect predictor through the mediation of satisfaction (Jarrett et al., 2024). Moreover, as a mediator, ATT positively influences repurchase intentions, driven by the need for uniqueness and social approval (Li et al., 2023). However, the evolution of ATTs from initial adoption to post-adoption requires further examination to understand their trajectory throughout the sustainable consumption journey. Environmental concerns have been found to impact perceived usefulness, perceived ease of use, satisfaction, and green loyalty (Li and Lin, 2022). Values such as green altruism and biospheric values also positively moderate the relationships between post-adoption perceptions and continued use (Puntiroli et al., 2022). Notably, Peters et al. (2018) discovered that individuals are more likely to maintain sustainable behaviors if they initially adopt them for environmental reasons, aligning with self-determination theory, which suggests that intrinsic motivations are more potent and enduring.

### 2.4 Pro-environmental self-identity

Theory of planned behavior has been expanded to include PESI as a crucial determinant of behavior, enhancing its predictive accuracy (Sparks and Guthrie, 1998). Self-identity, which refers to the label individuals assign to themselves, is influenced by personal motivations and social dynamics, including the expectations of others and the roles individuals assume (Bem, 1967; Fekadu and Kraft, 2001). In the current research, a pro-environmental identity is characterized as a persistent self-perception of interdependence with nature, indicating the degree to which an individual integrates environmentalism into their core identity (Lavuri et al., 2023). Thus, PESI is an individual’s self-conception that incorporates pro-environmental actions and is propelled by the drive for self-expression (Paquin and Keating, 2017). This self-identity is a robust predictor of behavior across a variety of situations, including sustainable consumption, and is considered a more reliable determinant than ATT, SN, and PBC (Whitmarsh and O’Neill, 2010). Within the realm of environmental behavior, individuals with a strong PESI are likely to view themselves as eco-friendly, influencing their decisions to develop or reinforce their self-concept (Van der Werff et al., 2013).

The connection between identity and behavior is well-established, with consumption behaviors and the adoption of new products being tied to identity (Mazhar et al., 2022). Research has established that self-identity is a crucial determinant of behavior, surpassing even the predictive power of the TPB variables, especially when it comes to environmentally friendly actions (Fekadu and Kraft, 2001). Moreover, various studies have verified that self-identity directly accounts for pro-environmental behaviors, as evidenced in studies on recycling intentions (Dermody et al., 2015) and a spectrum of pro-environmental behaviors such as waste reduction and eco-shopping (Whitmarsh and O’Neill, 2010). Dermody et al. (2015) indicated that PESI significantly impacts sustainable consumption, and in some cases, identity can even override ATT when role identity dictates certain behaviors, regardless of personal feelings towards those behaviors. More recent research indicates that PESI predicts intentions to protect the environment (Lavuri et al., 2023) and is positively related to individual consumer behavior and civic behavior regarding green food and beverage choices (Wang, 2016).

### 2.5 Habit

HABs are characterized as ingrained patterns of behavior that have turned into automatic reactions to specific cues and are functional in obtaining certain goals or end states (Verplanken and Aarts, 1999). This automaticity is not merely the repetition of actions but also the level of automaticity within consistent settings, which reduces the cognitive effort required to perform the behavior (Klöckner, 2013). This suggests that existing research on SCB may tacitly assume that actions are largely governed by cognitive deliberations. However, this assumption may not hold true for the ongoing phase of SCB, where cognitive assessments might diminish, allowing HABs to become a key driver of actions (Limayem et al., 2007). The initial uptake of a behavior influences its persistence, but this relationship is not straightforward. Instead, the link between adoption and persistence is mediated by post-adoption evaluations and the development of HABs. Favorable post-adoption evaluations reinforce the behavior, leading to the formation of HABs. As HABs become stronger, they reduce the need for conscious deliberation, making the behavior more automatic and less dependent on cognitive assessments (Wood, 2016). This process is supported by empirical evidence showing that HABs significantly influence continuance intention (Amoroso and Lim, 2017) and can moderate the relationships between purchase intention and perceived value, trust, and satisfaction (Hsu et al., 2015). Furthermore, the stronger the HAB, the less significant post-adoption evaluations become in explaining persistence, as the behavior becomes ingrained over time (Verplanken and Orbell, 2003). This suggests that while initial cognitive assessments are important for behavior adoption, the development of HABs is crucial for sustaining these behaviors over time. This research delves into the role of HABs and suggests strategies for fostering green HABs, which are pivotal for the enduring nature of consumer behaviors (Ghaffar and Islam, 2024).

### 2.6 Theoretical framework

The escalating concern for environmental sustainability has led to a significant shift in consumption patterns, with SCB emerging as a pivotal approach to mitigate the advers effects of traditional consumption HABs (Kaufmann et al., 2012). The theoretical framework for this study is anchored in the TPB, which posits that ATT, SN, and PBC as mediating variables (Ajzen, 1991), are the principal determinants of CBI (Qi and Ploeger, 2021). This study extends the TPB by incorporating PAE, EP, HAB, and PESI to enhance the understanding of the CBI among Chinese consumers who have had post-adoption experience with green products.

HAB is considered as a mediating variable as well, capturing the automatic response tendencies acquired through repeated behavior, which can significantly influence an individual’s PBC and CBI over time (Honkanen and Young, 2015). As sustainable behaviors become habitual, the need for conscious decision-making decreases, leading to a seamless integration of SCB into daily life.

**Figure 1:**
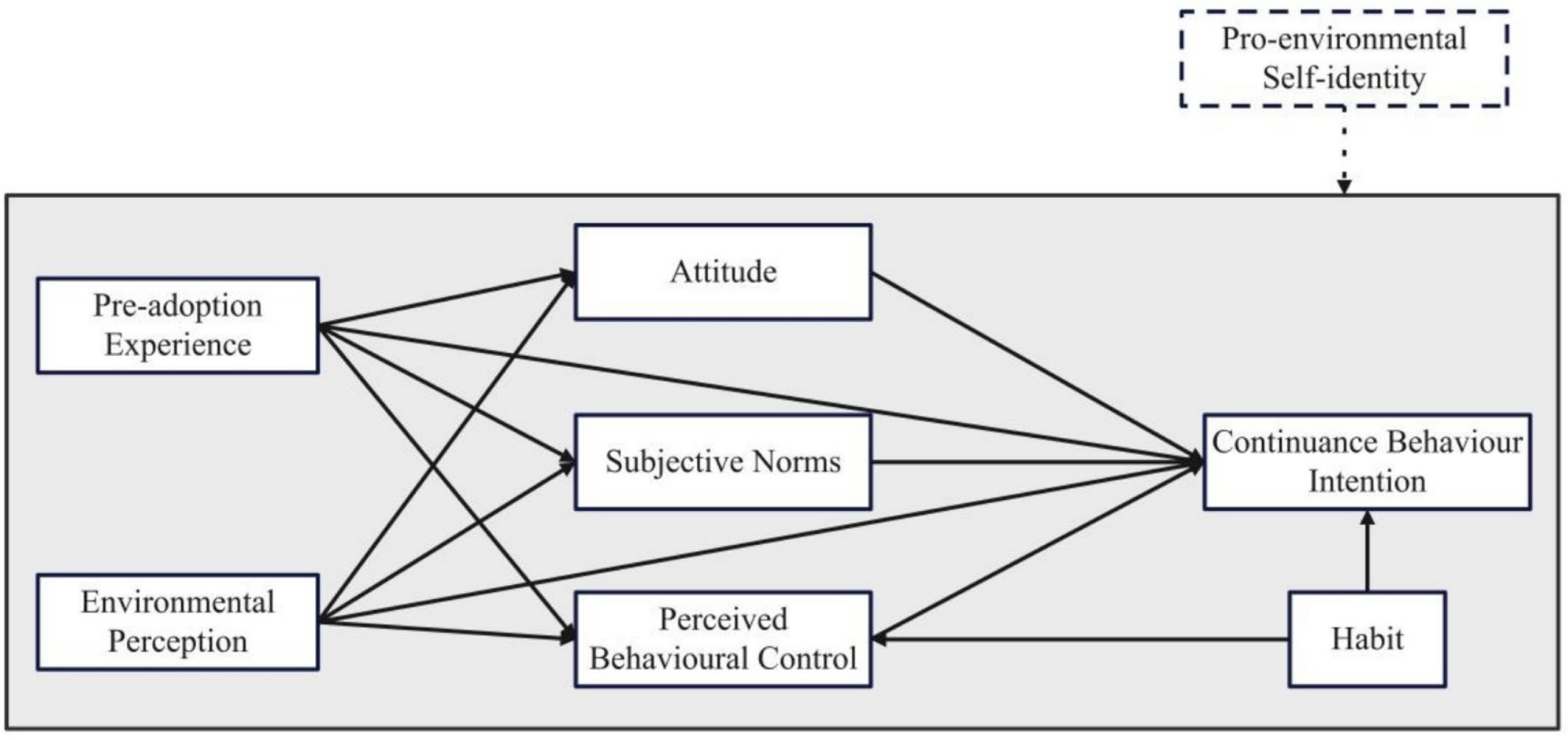
Proposed research model. The shaded area represents the model of continuance behavior intention proposed in this study, while the dashed-line box represents the moderating variables proposed in this study.

The research model proposed PESI as a moderating variable, reflecting the extent to which an individual’s self-concept is intertwined with pro-environmental actions (Dermody et al., 2015). PESI is expected to intensify the relationships between PAE, EP, ATT, SN, PBC, and CBI, as individuals with a robust environmental identity are more likely to maintain SCB due to the alignment with their self-concept (Fekadu and Kraft, 2001). This study aims to contribute to the literature by offering a more nuanced understanding of the factors influencing the continuance of SCB and by addressing the research gap in the post-adoption stage of sustainable consumer journey. Figure 1 presents the proposed research model in this study.

## 3 Hypotheses

This study is geared towards exploring the critical drivers that impact sustainable consumer behavior. Additionally, this study has investigated the potential differences in sustainable consumer intentions and behaviors moderated by PESI. Therefore, the 14 hypotheses are generated based on the statement below.

PAE is conceptualized through the lens of economic satisfaction, with a particular emphasis on consumers’ positive responses to price discounts during their initial encounters with green products (Chang et al., 2024). Research indicates that for price-sensitive segments, especially within the Chinese market, these favorable economic experiences, such as perceiving enhanced value through discounts, play a pivotal role in lowering the perceived financial barriers associated with trial and elevating the overall appraisal of sustainable offerings (Li and Kim, 2024). Grounded in empirical studies, this perspective aligns with the view that pre-adoption experiences constitute formative evaluations rooted in early economic interactions (Costa et al., 2021; Si et al., 2022). The more positive the PAE, the more likely it is to result in a positive ATT towards green products. Therefore, this study proposes the following first hypothesis:

*H1*: ATT is positively correlated with PAE.

PAE can significantly shape an individual’s perception of what is socially acceptable or expected, thereby influencing SN (Chang and Zhu, 2011). Positive pre-adoption experiences with green products can lead to the perception that society values environmental sustainability, thus influencing SN (Arli et al., 2018). When individuals believe that their groups endorse pro-environmental actions, they have a tendency to feel a sense of social pressure to conform to these expectations through their consumption choices (Chen and Chang, 2012). Research has shown that hands-on experience with green products can enhance their appeal and acceptance among consumers, which in turn can influence perceptions of social norms regarding environmental friendliness (Alruwaie, 2014). Drawing from the notion that PAEs shape social perceptions, this study proposes the second hypothesis:

*H2*: SN is positively correlated with PAE.

PAE is a critical factor that shapes an individual’s PBC regarding green products (Chang et al., 2024). PBC refers to the degree to which individuals feel they have the ability and resources to engage in sustainable consumption behaviors. Research has shown that individuals who have had positive PAEs with green products tend to feel capable of making sustainable consumption choices, which is a key component of PBC (Kapoor and Dwivedi, 2020). Lin et al. (2021) has found that PBC serves as a mediator between ATTs and intentions toward responsible environmental behavior in the marine context. This suggested that as individuals gain more positive experiences with green products, their perceived behavioral control increases, which in turn influences their intentions to engage in sustainable behaviors (Lin et al., 2021). Additionally, the research on cultural values and technology PAE provides insight into how different cultural backgrounds might influence the relationship between ATT, SN, and PBC, which can be relevant for understanding the broader implications of PAE on PBC (Li et al., 2023). Considering the empowering nature of positive PAEs on PBC, this study introduces the third hypothesis:

*H3*: PBC is positively correlated with PAE.

The impact of PAE on continuance intention is another critical aspect of sustainable consumer behavior (Gupta et al., 2020). When consumers perceive tangible economic benefits, it not only reduces the financial perceived risk of repeat purchases but also establishes a practical foundation for sustained engagement with green products (Si et al., 2022). This mechanism is particularly relevant in the context of green consumption, where initial cost concerns often act as a barrier to adoption. It suggests that consumers’ cognitive perceptions in a brand will influence their affective feelings or attitudes towards the brand and finally result in the behavioral purchasing intention (Huang and Chen, 2021). This intention is based on the practical benefits and satisfaction derived from the initial experiences, which can foster loyalty and repeat purchasing behavior (Liébana-Cabanillas et al., 2021). Additionally, empirical evidence suggests that positive evaluations of economic utility during trial phases can translate into a stronger motivation for long-term repurchase (Li and Kim, 2024), as consumers become confident in the functional and economic value of sustainable options. Hence, this study suggests the fourth hypothesis:

*H4*: CBI is positively correlated with PAE.

EP refers to the extent of individuals’ awareness and concern regarding environmental issues. The relationship between EP and ATT is underpinned by the premise that those with heightened environmental awareness and concern are more inclined to foster positive ATTs towards sustainable consumption (Tanner and Wölfing Kast, 2003). The degree to which individuals perceive environmental issues can significantly shape their ATTs towards green products, as this perception is linked to the adoption of sustainable behaviors (Cherian and Jacob, 2012). Additionally, research has consistently shown that EP is a key predictor of pro-environmental behavior, including the adoption of green products (Truelove and Gillis, 2018). When individuals recognize the importance of environmental sustainability, they are more likely to view green products positively, appreciating their potential to reduce environmental harm (Moser, 2015). This positive ATT can be further reinforced by the experience of using green products, which can demonstrate their effectiveness and align with personal values of sustainability (van Riper et al., 2019). These hypotheses are supported by a growing body of literature that emphasizes the role of EP in shaping both ATT and SN related to green product consumption (Kharat et al., 2017). By raising awareness of environmental issues and promoting the value of sustainable behaviors, individuals can be motivated to adopt greener consumption patterns, which can contribute to the broader goal of sustainable development. Given the correlation between environmental awareness and positive ATTs, this study formulates the fifth hypothesis:

*H5*: ATT is positively correlated with EP.

EP can be a powerful motivator for adopting sustainable behaviors, as individuals often seek to align their actions with the expectations of their social group (Tian and Liu, 2022). When individuals perceive environmental issues as acute and widespread, they are more likely to infer that these concerns are also salient within their social referent groups (Farrow et al., 2017). This inference transforms a personal cognition into a descriptive social norm (what is commonly done), which in turn strengthens the injunctive social norm (what is approved or expected by others) (Arli et al., 2018). The study by Saltik and Akova (2024) provides direct empirical support for this pathway, demonstrating that tourists’ perceptions of environmental issues significantly increased the pressure they felt from subjective norms. This is because a shared perception of an environmental problem creates a common ground for social expectations to emerge and be internalized. Acknowledging the pressure of social expectations in the context of environmental issues, this study proposes the sixth hypothesis:

*H6*: SN is positively correlated with EP.

Beyond shaping social expectations, a heightened awareness of environmental challenges can empower individuals by enhancing their perceived capability to act. Knowledge and awareness are key components of self-efficacy, a core dimension of PBC. Individuals with a strong environmental perception are likely to be more informed about environmental issues and sustainable alternatives (Gardner and Abraham, 2010). This knowledge reduces uncertainty and increases their confidence in navigating potential barriers (e.g., identifying green products, understanding their benefits), thereby strengthening their belief in their ability to perform green purchasing behaviors (De Leeuw et al., 2015). This conceptual link is further reinforced by empirical evidence from other domains, such as the finding that environmental trust significantly influences behavioral intentions (Chen, 2017). Hence, the seventh hypothesis is hereby presented within the scope of this investigation:

*H7*: PBC is positively correlated with EP.

The perception of environmental issues can directly influence an individual’s intention to continue their sustainable consumption behaviors, known as continuance behavior intention. When individuals are more aware of the environmental consequences of their actions, they are likely to maintain behaviors that they perceive as beneficial to the environment (Si et al., 2022). This continued intention can be seen as a long-term commitment to sustainable consumption, driven by a deep-seated concern for environmental well-being. Research has indicated that environmental concern is a key predictor of continuance intention, including the adoption of green products (Carfora et al., 2019). Recognizing the direct influence of environmental concern on sustainable consumption, this study presents the eighth hypothesis:

*H8*: CBI is positively correlated with EP.

A positive ATT towards green products and their benefits is a crucial factor in forming a CBI to engage in sustainable consumer behavior. When consumers hold favorable ATTs towards green products, they are more likely to express an intention to continue purchasing such products. This ATT reflects their overall evaluation and liking for green products, which can drive their future behavior (Steg et al., 2014). Extant literature supports the notion that a positive ATT is a significant predictor of intention (Honkanen and Young, 2015). Given the predictive power of ATT on behavioral intention, this study proposes the ninth hypothesis:

*H9*: CBI is positively correlated with ATT.

SN can significantly influence CBI of sustainable consumer behavior (Tripathi et al., 2021). When individuals feel social pressure to conform to sustainable consumption patterns, they are more likely to express an intention to continue such behaviors (Si et al., 2022). This intention can be seen as a response to the perceived expectations of others, including family, friends, and the broader community. The role of SN in shaping pro-environmental behaviors has been well-documented in the literature, emphasizing the influence of social factors on individual intentions (Mouakket, 2015). Considering the social pressures that drive sustainable consumption, this study introduces the tenth hypothesis:

*H10*: CBI is positively correlated with SN.

PBC plays a crucial role in influencing CBI toward sustainable consumer behavior. When individuals feel a sense of control over their ability to engage in sustainable practices, they are more likely to express a strong intention to continue those behaviors (Yan et al., 2024). This sense of control can stem from previous experiences, knowledge, and resources that empower individuals to make environmentally friendly choices. Research has consistently shown that higher levels of perceived behavioral control are associated with increased intentions to engage in sustainable behaviors (Ajzen, 2002). Given the significance of perceived control in sustainable consumer intentions and behaviors, this study proposes the eleventh hypothesis:

*H11*: CBI is positively correlated with PBC.

HAB, defined as automatic behavioral patterns triggered by contextual cues, fundamentally alters the cognitive calculus of performing a behavior. As green purchasing behaviors become habitual through repetition, the cognitive effort and resources required to execute them diminish significantly (Ouellette and Wood, 1998; Verplanken and Orbell, 2003). This process of automatization directly reduces perceived barriers and increases the perceived ease of performing the behavior, which is a core component of PBC (Pollard, 2006). Empirical evidence supports this link. For instance, Demir et al. (2023) found habits to be strongly associated with perceived behavioral control within the domain of mobile phone use while driving. The researchers posit that habitual engagement reinforces individual’s sense of mastery over the behavior. Recognizing the habitual nature of behaviors and its impact on perceived control, this study presents the twelfth hypothesis:

*H12*: PBC is positively correlated with HAB.

HAB, referred as the automatic performance of behaviors due to prior learning, significantly influences continuance intention by reducing the cognitive effort required to perform a behavior (Ouellette and Wood, 1998; Verplanken and Aarts, 1999). Empirical studies have consistently shown that habit is a strong predictor of continuance intention. For instance, Liao et al. (2006) demonstrated that habit, perceived usefulness, and trust collectively determine online purchase behavior. Amoroso and Lim (2017) found a strong relationship between habit and continuance intention, mediated by perceived switching costs. Hsu et al. (2015) further revealed that habit moderates the relationships between purchase intention and perceived value, trust, and satisfaction. Given the automaticity of HABs in shaping future intentions, this study introduces the thirteenth hypothesis:

*H13*: CBI is positively correlated with HAB.

PESI is theorized to be a critical boundary condition that intensifies the relationships within the model by mitigating the value-action gap. Individuals with a high PESI have internalized environmentalism as a core aspect of their self-concept (Clayton, 2003; Dermody et al., 2015). This strong identity ensures a high degree of cognitive consistency between their environmental values, attitudes, their behavioral intentions (Chwialkowska et al., 2020). For them, the pathways from cognitive drivers (PAE, EP) and social influences (SN) to intention (CBI) are likely to be stronger and more reliable, as their actions are driven by a desire to act in accordance with their self-view. Conversely, for individuals with low PESI, these relationships are likely weaker. Their intentions are more susceptible to external, situational factors (e.g., cost, convenience) rather than internalized values, making the links between their perceptions, attitudes, and intentions more volatile and less predictable. Furthermore, the literature provides a specific basis for its moderating role. Research on abnormally shaped foods has shown that PESI can strengthen the relationship between product attributes (abnormality) and purchase intention (Loebnitz and Grunert, 2015). More broadly, Carfora et al. (2017) explicitly tested and discussed the moderating effects of PESI within an extended TPB model, providing a direct theoretical and empirical foundation for our hypothesis. Building on this, this study proposes the fourteenth hypothesis:

*H14*: PESI moderates the relationships of PAE to ATT, PAE to SN, PAE to PBC, PAE to CBI, EP to ATT, EP to SN, EP to PBC, EP to CBI, ATT to CBI, SN to CBI, PBC to CBI, HAB to PBC, and HAB to CBI.

## 4 Methodology

### 4.1 Demographic results

The proportional-quota non-probability sampling method was employed, based on gender distribution in China according to data from the National Bureau of Statistics of China (NBSC, 2023). Participants were recruited through an online panel via the survey platform https://www.wjx.cn, and were included if they had prior experience in purchasing or using green products (i.e., post-adoption experience). Data were collected via a questionnaire that included demographic questions and surveys on green products. Prior to participation, respondents were provided with a written informed consent statement and were explicitly informed about the criteria defining green products: (1) certification under USDA Organic, EU Eco-Label, or China Organic Product standards (GB/T 19630–2019); (2) production without synthetic pesticides, genetic modification, or chemical fertilizers; (3) packaging with ≥80% biodegradable/recyclable materials.

Table 1 presents the demographic profile of respondents. The sample was initially expected to have a sampling error of ±5% at a 95% confidence level, resulting in a target sample size of n = 588. Data were collected via a questionnaire that included demographic questions and surveys on green products. Of the 588 valid responses, 53.4% were male and 46.6% were female, closely reflecting the NBSC data (males accounting for 51.15% and females for 48.85%). This alignment ensured that the sample was representative of the target population. Regarding age distribution, 1.36% of respondents were under 18 years old, 15.99% were aged 18–25 years, 34.69% were aged 26–35 years, 26.87% were aged 36–45 years, 20.75% were aged 46–55 years, and 0.34% were over 56 years old. Furthermore, 210 respondents identified themselves as having a PESI, indicating a strong willingness to purchase green products. Conversely, 378 respondents did not perceive themselves as having a PESI, either due to a lack of strong intent to purchase green products or indifference to the environmental attributes of such products. Representative items include organic vegetables, fair-trade coffee, and snacks in compostable packaging. This operationalization aligns with FAO/WHO Codex Alimentarius guidelines for organic foods and prior studies on green consumption (Mazhar et al., 2022; Zhang et al., 2024), maintaining ecological validity for Chinese consumers. In terms of educational attainment, 55.44% of respondents held a bachelor’s degree, while 23.13% had incomplete university studies. Regarding gross monthly income, 10.88% earned less than ¥3,000, 20.86% earned between ¥3,001 and ¥6,000, 57.95% earned between ¥6,001 and ¥10,000, and 3.06% reported earning more than ¥10,000.

**Table 1:** Demographic characteristics of the sample (*N* = 588).

Variable	Category	Frequency	Percent (%)	Cumulative percent (%)
Gender	Male	314	53.40	53.40
	Female	274	46.60	100.00
Age (years)	<18	8	1.36	1.36
	18–25	94	15.99	17.35
	26–35	204	34.69	52.04
	36–45	158	26.87	78.91
	46–55	122	20.75	99.66
	>56	2	0.34	100.00
Monthly income (¥)	<3,000	64	10.88	10.88
	3,001–6,000	123	20.86	31.74
	6,001–10,000	341	57.95	89.69
	>10,000	18	3.06	92.75
Education level	Primary	2	0.34	0.34
	Secondary	70	11.90	12.24
	Incomplete College	64	10.88	23.12
	Bachelor’s	326	55.44	78.56
	Master’s	108	18.37	96.93
	PhD	18	3.07	100.00
PESI group	With PESI	210	35.71	35.71
	Without PESI	378	64.29	100.00

### 4.2 Measurement scale development

The measurement scales for PAE, EP, ATT, SN, PBC, HAB, and CBI constructs were adapted from the studies of Costa et al. (2021), Mónus (2020), Paul et al. (2016), Hsu et al. (2015), and Suhartanto et al. (2021), respectively (see Appendix A). Responses were scored on a five-point Likert scale ranging from 1 = strongly disagree to 5 = strongly agree. The PAE scale focuses specifically on price satisfaction as it represents a critical initial encounter for Chinese consumers, for whom discounts are a key trigger in the trial phase of green products preceding sustained adoption. The HAB scale focuses on the routine of “shopping at stores that sell green products.” This operationalization is predicated on the context-dependent nature of habit formation, where environmental cues, such as specific retail environments, can trigger automatic behavioral sequences. Moreover, for the CBI items (e.g., “I have the intention to repurchase the green products in the future”), participants were instructed to respond with reference to the same specific green product they now regularly purchase, which they had identified at the beginning of the survey. PESI was measured using a single binary item: “I have a pro-environmental self-identity” (yes/no), adapted from McCright and Dunlap (2015). Respondents who selected “yes” were classified as having a PESI (coded as 0), indicating a strong willingness to purchase green products, while those who selected “no” were classified as lacking a PESI (coded as 1). To ensure reliability and validity across cultural and linguistic contexts, the measurement scale was translated into Chinese through a systematic process involving initial translation, back-translation, review for linguistic and cultural equivalence by marketing professors, pilot testing with marketing students, refinement based on feedback, a second back-translation for meaning equivalence, and final adjustments to produce the finalized Chinese version.

## 5 Results

### 5.1 Measurement model

Descriptive statistics were calculated using SPSS 26 (version 26) (Table 2). Standard deviations ranged from 0.732 to 1.118 across all constructs, exceeding the 0.5 threshold for adequate variability (Hair et al., 1998), with means between 3.105 and 4.588 on a 5-point scale (*N* = 588).

**Table 2:** Descriptive statistics of measurement items.

Construct	Variable	Mean	Std. dev.	Min	Max
ATT	ATT1	4.116	0.889	1	5
	ATT2	4.350	0.823	1	5
	ATT3	4.313	0.872	1	5
CBI	CBI1	3.871	0.903	1	5
	CBI2	3.432	1.086	1	5
	CBI3	3.782	0.959	1	5
EP	EP1	4.109	1.045	1	5
	EP2	3.993	1.038	1	5
	EP3	4.588	0.732	1	5
	EP4	4.146	0.913	1	5
	EP5	4.041	1.020	1	5
HAB	HAB1	3.105	1.101	1	5
	HAB2	3.364	1.011	1	5
	HAB3	3.173	1.067	1	5
PAE	PAE1	3.735	1.023	1	5
	PAE2	3.656	1.058	1	5
	PAE3	3.782	1.048	1	5
PBC	PBC1	3.837	0.998	1	5
	PBC2	3.840	0.993	1	5
	PBC3	4.048	0.947	1	5
	PBC4	3.646	1.062	1	5
	PBC5	3.565	1.117	1	5
	PBC6	3.507	1.104	1	5
SN	SN1	3.714	1.064	1	5
	SN2	3.721	1.053	1	5
	SN3	3.772	1.044	1	5
	SN4	3.878	0.973	1	5

To address common method bias, Harman’s single-factor test was conducted using SPSS (version 26). The eigenvalue of the first factor accounted for 46.707% of the variance (Table 3), which is less than the threshold of 50%. This result indicates that common method bias is not present in this study, and the analysis is free from measurement error (Hair et al., 1998; Hair et al., 2019).

**Table 3:** Common method bias test.

Total	% of Variance	Cumulative %
13.078	46.707	46.707

The convergent validity of the measurement model is assessed through three criteria: (1) factor loadings should be significant and exceed the minimum threshold of 0.5, (2) composite reliability (CR) values should be greater than 0.6 (Fornell and Larcker, 1981), and (3) average variance extracted (AVE) values should surpass 0.5 (Chin, 1998). Additionally, Cronbach’s alpha is used as an indicator of the internal consistency reliability of a scale, with an acceptable threshold of 0.6 or higher (Nunnally and Bernstein, 1994). As shown in Table 4, most factor loadings are above 0.8, with the highest value reaching 0.951. These results indicate that the model is reliable, as all item factor loadings exceed 0.6, and the AVE values are greater than 0.6, with the highest AVE value being 0.882. This demonstrates that the measurement model exhibits a strong convergent effect. Furthermore, all Cronbach’s alpha values are above 0.7, and the CR values for the constructs exceed 0.9, indicating a desirable level of internal consistency among the constructs.

**Table 4:** Factor loading, Cronbach’s alpha, CR, AVE of constructs.

Construct	Item	Factor Loading	Cronbach’s alpha	CR	AVE
ATT	ATT 1	0.930	0.933	0.957	0.882
	ATT 2	0.944			
	ATT 3	0.944			
CBI	BI 1	0.882	0.858	0.914	0.779
	BI 2	0.842			
	BI 3	0.922			
EP	EP 1	0.803	0.844	0.889	0.616
	EP 2	0.796			
	EP 3	0.719			
	EP 4	0.798			
	EP 5	0.805			
HAB	HAB 1	0.91	0.901	0.938	0.835
	HAB 2	0.894			
	HAB 3	0.936			
PAE	PAE 1	0.916	0.860	0.912	0.776
	PAE 2	0.898			
	PAE 3	0.826			
PBC	PBC 1	0.811	0.914	0.933	0.701
	PBC 2	0.853			
	PBC 3	0.821			
	PBC 4	0.883			
	PBC 5	0.831			
	PBC 6	0.821			
SN	SN 1	0.924	0.937	0.955	0.842
	SN 2	0.951			
	SN 3	0.944			
	SN 4	0.847			

To assess discriminant validity, the correlation coefficient between the square root of the average variance extracted (AVE) and all possible constructs is compared. The square root of the AVE must exceed the values of all potential constructs to establish discriminant validity. According to Table 5, all the square root values of the AVE are higher than those of the other constructs, indicating strong discriminant validity.

**Table 5:** Simple correlation matrix and discriminant validity.

Construct	ATT	CBI	EP	HAB	PAE	PBC	SN
ATT	0.939						
CBI	0.614	0.883					
EP	0.590	0.570	0.785				
HAB	0.388	0.619	0.348	0.914			
PAE	0.397	0.347	0.391	0.324	0.881		
PBC	0.720	0.704	0.526	0.594	0.431	0.837	
SN	0.720	0.641	0.498	0.593	0.382	0.783	0.918

Additionally, the Heterotrait-Monotrait Ratio (HTMT) is employed to further examine discriminant validity. For the HTMT, values must be less than 1 to confirm validity (Henseler et al., 2015). Table 6 shows that all HTMT values in this study are below 1, reinforcing the discriminant validity of the measurement.

**Table 6:** Heterotrait-Monotrait ratio.

Construct	ATT	CBI	EP	HAB	PAE	PBC	SN
ATT							
CBI	0.685						
EP	0.664	0.660					
HAB	0.423	0.703	0.393				
PAE	0.417	0.383	0.440	0.353			
PBC	0.775	0.793	0.592	0.654	0.465		
SN	0.770	0.714	0.554	0.644	0.409	0.844	

### 5.2 The structural model

**Figure 2:**
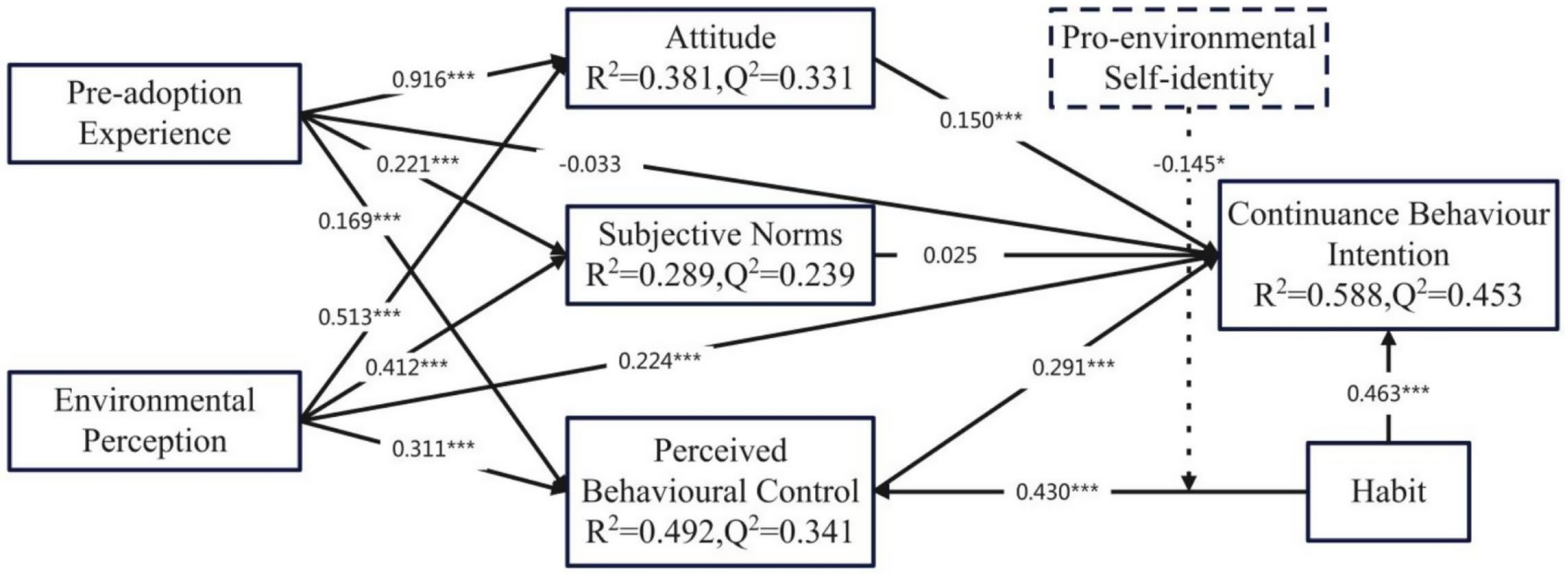
Structural model PLS results.

The predictive ability of the structural model can be assessed through R^2^ value of each endogenous construct that should be higher than 0.1 (Falk and Miller, 1992) and the Stone-Geisser test of predictive relevance (Q^2^), whose values are divided into 0.02, 0.15 and 0.35 as small, medium and large effects, respectively, (Geisser, 1975). According to Figure 2, the values of R^2^ (0.381, 0.588, 0.492 and 0.289) and Q^2^ (0.331, 0.453, 0.341 and 0.239) suggest that the structural model demonstrates strong predictive ability.

Table 7 shows the 13 research hypotheses are verified. H1 (0.196***), H2 (0.221***), H3 (0.169***), H5 (0.513***), H6 (0.412***), H7 (0.311***), H8 (0.224***), H9 (0.150***), H11 (0.291***), H12 (0.430***) and H13 (0.463***) are positive supported, while H4 (0.263) and H10 (0.637) are not positively supported.

**Table 7:** The *t*-value of research hypotheses and path coefficients.

No.	Research hypotheses	*t*-statistic	Path coefficients	*p*-value	Validated result
H1	PAE→ATT	4.867	0.196	0.000***	Supported
H2	PAE→SN	4.962	0.221	0.000***	Supported
H3	PAE→PBC	4.464	0.169	0.000***	Supported
H4	PAE→CBI	1.122	−0.033	0.263	Not Supported
H5	EP→ATT	11.884	0.513	0.000***	Supported
H6	EP→SN	10.867	0.412	0.000***	Supported
H7	EP→PBC	7.914	0.311	0.000***	Supported
H8	EP→CBI	6.175	0.224	0.000***	Supported
H9	ATT→CBI	3.166	0.150	0.002***	Supported
H10	SN→CBI	0.473	0.025	0.637	Not Supported
H11	PBC→CBI	5.577	0.291	0.000***	Supported
H12	HAB→PBC	10.921	0.430	0.000***	Supported
H13	HAB→CBI	11.819	0.463	0.000***	Supported

*p < 0.1; **p < 0.05; ***p < 0.01.

Considering the coefficients in Table 8, this study confirms a partial mediation effect of ATT on the paths of EP → ATT → CBI, and PAE → ATT → CBI, respectively; a partial mediation effect of PBC on the paths of EP → PBC → CBI, HAB → PBC → CBI, and PAE → PBC → CBI, respectively.

**Table 8:** Specific indirect effects.

Path	Path coefficients	*t*-statistic	*p*-value	Validated result
EP → ATT → CBI	0.077	3.102	0.002***	Supported
PAE → ATT → CBI	0.029	2.503	0.013***	Supported
EP → PBC → CBI	0.09	4.917	0.000***	Supported
HAB → PBC → CBI	0.125	4.535	0.000***	Supported
PAE → PBC → CBI	0.049	3.523	0.000***	Supported
EP → SN → CBI	0.01	0.474	0.636	Not supported
PAE → SN → CBI	0.005	0.465	0.642	Not supported

*p < 0.1; **p < 0.05; ***p < 0.01.

### 5.3 Moderation analysis

In order to test the moderating effect of PESI in the model, 588 respondents participated in the survey were divided into Group 1 = with PESI and Group 2 = without PESI. Before proceeding to multi-group analysis, we measured the invariability of the measurement models through MICOM (Rialp-Criado and Rialp-Criado, 2018). Following the approach of Sinkovics et al. (2016) and Moon (2021), the MICOM procedure comprises three steps: (1) configural invariance, (2) compositional invariance, (3) the equality of composite mean values and variances. Table 9 shows that the configurational and compositional are observed but the third one, so a partial measurement invariance can be established, which allows us to proceed with multi-group analysis.

**Table 9:** Configural invariance and Compositional invariance.

Construct	Configural invariance	Compositional invariance			Measurement invariance
		Original Correlation	Permutation *p*-values	Compositional invariance	
ATT	Yes	1.000	0.391	Yes	Partial
CBI	Yes	1.000	0.257	Yes	Partial
EP	Yes	0.999	0.462	Yes	Partial
HAB	Yes	1.000	0.613	Yes	Partial
PAE	Yes	0.996	0.106	Yes	Partial
PBC	Yes	1.000	0.162	Yes	Partial
SN	Yes	1.000	0.265	Yes	Partial

Table 10 presents the results of the hypotheses further developed on the moderating effect of PESI. The MGA results support H14 (the moderating effect of PESI) because the coefficients testing H12, being significant for both groups present significant differences among them at 90% of confidence.

**Table 10:** Causal hypotheses testing and multi-group comparison test results.

No.	Path	Path coefficients (GROUP1 – GROUP2)	*t*-statistic	*p*-value	Validated result
H1	PAE→ATT	0.027	0.296	0.767	Not supported
H2	PAE→SN	0.101	1.000	0.318	Not supported
H3	PAE→PBC	0.057	0.692	0.489	Not supported
H4	PAE→CBI	−0.072	1.108	0.268	Not supported
H5	EP→ATT	0.082	0.912	0.362	Not supported
H6	EP→SN	0.003	0.038	0.969	Not supported
H7	EP→PBC	0.046	0.596	0.552	Not supported
H8	EP→CBI	0.110	1.324	0.186	Not supported
H9	ATT→CBI	−0.084	0.789	0.431	Not supported
H10	SN→CBI	−0.107	0.870	0.384	Not supported
H11	PBC→CBI	0.039	0.339	0.734	Not supported
H12	HAB→PBC	−0.145	1.765	0.078*	Supported
H13	HAB→CBI	0.058	0.647	0.518	Not supported

*p < 0.1; **p < 0.05; ***p < 0.01.

## 6 Discussion

Aligned with previous studies (Granato et al., 2022; Zacher et al., 2023), the present study provides valuable insights into the factors influencing consumers continuance behavior intention to purchase green products, specifically focusing on consumers’ ATT, SN, PBC, PAE, EP, HAB and PESI. These variables all contributed positively to sustaining continuance behavior intention, underscoring the complexity and multi-dimensional nature of consumer behavior. The results are thoroughly examined in the following sections.

### 6.1 Levels of PAE, EP, ATT, SN, PBC, HAB toward CBI

The significant and positive relationships identified between PAE and its immediate outcomes of ATT, SN, and PBC are consistent with prior research. Specifically, the findings for H1 (0.196***), H2 (0.221***), and H3 (0.169***) underscore the foundational role of PAE in shaping the cognitive and normative antecedents of behavioral intention. These results align with studies by Chang et al. (2024) and Li and Kim (2024), which emphasize that initial positive experiences with green products significantly influence perceptions of feasibility, social acceptability, and personal control in sustainable consumption.

The strong relationship between EP and its downstream effects further highlights the importance of environmental awareness in driving pro-environmental ATTs and behaviors. The significant coefficients for H5 (0.513***), H6 (0.412***), and H7 (0.311***) suggest that individuals who are more aware of environmental issues tend to develop favorable ATTs, feel social pressure to engage in sustainable behaviors, and perceive greater control over their ability to act sustainably. These findings are in line with Truelove and Gillis (2018) and Gardner and Abraham (2010), who have demonstrated that heightened environmental awareness fosters pro-environmental intentions by aligning personal values with broader social and environmental goals. The results for H9 (0.150***) and H11 (0.291***) reaffirm TPB (Ajzen, 1991), emphasizing that ATTs and perceived control are critical predictors of behavioral intention. Moreover, the strong support for HAB as a determinant of both PBC (H12, 0.430***) and CBI (H13, 0.463***) suggests that routine and automaticity play a central role in sustaining green consumption behaviors over time. This finding aligns with Wood (2016) and Ouellette and Wood (1998), who argue that HABs reduce cognitive load, enabling consistent performance of sustainable behaviors even in the absence of deliberate effort.

Despite the broad support for the model, the lack of positive support for H4 (0.263) and H10 (0.637) warrants careful examination. The nonsignificant relationship between PAE and CBI challenges the assumption that early positive experiences directly translate into long-term behavioral intentions. One possible explanation is that the influence of PAE on CBI is mediated through intermediary constructs such as ATT, SN, or PBC, rather than exerting a direct effect. This aligns with previous findings by Lin et al. (2021), who noted that PAEs shape sustainable behaviors primarily through changes in cognitive ATTs and perceived efficacy. This finding aligns with behavioral economics principles, because PAEs function as “mental accounts” (Thaler, 1985) where sunk costs (e.g., trial investments) prime cognitive ATTs but require policy-driven incentives (e.g., green subsidies) to convert into CBI. Furthermore, as Kapoor and Dwivedi (2020) suggest, the complexity of consumer decision-making in sustainability contexts may dilute the direct impact of initial experiences on long-term intentions, especially when external factors (e.g., product accessibility or cost) intervene. Here, carbon taxes could recalibrate cost perceptions, making green products economically salient and bridging the PAE-CBI gap.

Besides, the lack of support for the direct effect of SN on CBI raises intriguing questions about the role of social pressure in driving sustainable consumption. While SN are widely recognized as significant predictors of intention in the TPB framework, their influence may be contingent on specific contextual factors. In the context of green products, intrinsic motivations such as ATTs and HABs may outweigh external social pressures in determining continuance behavior. This finding is consistent with Mouakket (2015), who noted that SN are less impactful in contexts where personal values strongly align with the behavior in question. Additionally, the strong habit-forming nature of green consumption (evidenced by H12 and H13) may render social pressure less relevant over time, as consumers internalize sustainable behaviors as part of their identity and routine. From an identity economics lens (Gomes et al., 2022), this implies HABs reduce the “cognitive tax” of deliberate decision-making, enabling cost-efficient repetition of sustainable choices.

### 6.2 Mediation effects of LBE, LBI, LBQ and indirect effect of ETN

The partial mediation of ATT in the EP → ATT → CBI pathway demonstrates that EP significantly shapes continuance intentions both directly and indirectly through positive ATTs. This finding underscores that individuals with heightened environmental awareness are more likely to develop favorable ATTs toward green products, which subsequently enhance their intention to continue using these products. This supports Ajzen’s (1991) TPB, emphasizing ATT as a pivotal predictor of intention, and aligns with Tanner and Wölfing Kast’s (2003) assertion on the role of environmental awareness in fostering sustainable ATTs. Similarly, the mediation effect of ATT in the PAE → ATT → CBI pathway illustrates the pivotal role of cognitive and affective evaluations in translating PAEs into sustainable behavior intentions. Although not directly influencing CBI, PAEs contribute to attitudinal shifts that drive behavioral outcomes. This result reinforces the findings of Lin et al. (2021), which emphasize the importance of experiential learning in shaping ATTs that promote sustainable consumption.

The confirmation of PBC as a partial mediator in the EP → PBC → CBI pathway reveals that environmental awareness empowers individuals with a sense of control over their sustainable choices, thereby enhancing their continuance intention. This perceived control mirrors “nudge” mechanisms (Thaler and Sunstein, 2008), as policies like green subsidies lower financial barriers, transforming environmental awareness into economically viable actions by altering PBC’s cost–benefit calculus. This finding echoes Gardner and Abraham’s (2010) perspective on the empowering role of environmental awareness in fostering perceived efficacy and decision-making confidence in sustainability contexts. Besides, the mediation effect of PBC in the HAB → PBC → CBI pathway highlights that habitual sustainable practices bolster individuals’ perceived ability to maintain these behaviors, thus strengthening their intention to continue. This result aligns with Ouellette and Wood’s (1998) framework on HAB as a driver of behavior and suggests that automaticity in HABs not only sustains sustainable behaviors but also enhances confidence in executing them. Furthermore, the pathway PAE → PBC → CBI elucidates how PAEs indirectly influence continuance intentions by fostering a sense of control. This finding complements Ajzen’s (1991) TPB, emphasizing PBC as a critical determinant of behavioral intention, and underscores the importance of experiential engagement in empowering consumers toward sustainability.

### 6.3 Moderation effect of PESI

The analysis revealed a significant moderating effect of PESI on the relationship between HAB and CBI. Contrary to what might be intuitively expected, the positive effect of habit on intention was significantly stronger for consumers with low PESI. This finding provides a critical nuance to our understanding of how identity and automaticity interact. For consumers with high PESI, their intention to maintain green consumption is primarily and consistently driven by their deep-seated internal identity and values. Consequently, the additional effect of habitual automaticity on their intention is relatively weaker; their behavior is already strongly motivated by who they are. Conversely, for consumers with low PESI, who lack this powerful internal driver, the automaticity generated by habit becomes a dominant and crucial mechanism for sustaining behavioral intention. This suggests that for individuals not yet strongly identified as “green,” fostering habitual behaviors is an essential strategy to ensure the maintenance of sustainable consumption, effectively compensating for lower levels of internal motivation. This finding aligns with the dual-process models in behavioral economics (Thaler, 1985). High-PESI individuals engage in more deliberate, value-driven decision-making, while low-PESI individuals are more influenced by automatic, heuristic processes. Therefore, intervention strategies can be tailored: for high-PESI “deliberators,” policies can focus on providing value-congruent information, while for low-PESI “heuristic actors, “policies should focus on making green choices the easy, default, and habitual option through nudges and subsidies. These findings contribute to a deeper understanding of the boundary conditions of habit and highlight the need for multi-faceted interventions that address both deliberative and automatic pathways to sustainability.

## 7 Conclusion and implications

This study provides critical insights into the complex interplay of factors shaping consumers’ CBI toward green product consumption. By examining antecedents of PAE, EP, ATT, SN, PBC, HAB and PESI, the findings underscore the multidimensional nature of sustainable consumer behavior. The results confirm the pivotal roles of cognitive, normative, and habitual mechanisms in driving environmentally conscious behaviors. Moreover, the significant relationships between PAE and its outcomes (ATT, SN, and PBC) underline the importance of early positive experiences in shaping consumers’ perceptions and normative frameworks. Similarly, EP emerged as a crucial determinant, fostering favorable ATTs, social pressures, and a sense of control, all of which support stronger green consumption intentions.

However, the study also identifies notable nuances and unexpected findings. The non-significant direct relationship between PAE and CBI suggests that the influence of PAEs may be mediated through intermediary constructs like ATT or PBC rather than exerting a direct effect. Additionally, the limited impact of SN on CBI highlights the potential dominance of intrinsic motivations, such as personal values and HABs, over external social pressures in shaping green behaviors. Furthermore, the significant yet counterintuitive moderating role of PESI suggests that individuals with a strong pro-environmental identity may engage in deliberate decision-making processes rather than relying on habitual behaviors. These findings add depth to our understanding of the dynamic relationships among identity, HAB, and intention in sustainable consumption contexts.

The study has dual implications: theoretical and practical. Theoretically, this research extends TPB by integrating PESI as a moderating factor, offering a richer understanding of the role of self-concept in sustainable behaviors. By revealing how PESI interacts with EP, ATT, HAB, and CBI, the study highlights the importance of incorporating identity constructs into behavioral models to account for long-term sustainability engagement. Additionally, the findings reinforce the centrality of HAB in driving consistent green behaviors, suggesting that future research should investigate the dynamic interplay between ATTs, norms, and HABs over time.

Practically, the findings offer actionable insights for marketers and policymakers, with strategies tailored to the specific psychological mechanisms identified in this study. Marketing practitioners can strengthen the alignment between green products and consumers’ self-concepts by incorporating identity salience cues in campaigns, thereby activating PESI and reinforcing the connection between identity and behavior. For instance, framing purchasing decisions as reflections of pro-environmental values through messaging such as “Choose what aligns with who you are.” To foster habitual green consumption, brands may optimize choice architecture by placing eco-friendly products in high-visibility locations and introducing contextual cues, such as branded reminders for reordering sustainable staples, to reduce decision friction and promote automaticity (Li, 2025). Enhancing PAE through low-risk trial opportunities, such as free samples or money-back guarantees for green products, can also shape favorable ATT and PBC, laying a foundation for sustained engagement (Ketelsen et al., 2020). For policymakers, interventions aimed at fostering PESI may include community-based initiatives such as local environmental clean-up drives, which strengthen group identification with pro-environmental roles, and self-affirmation exercises that prompt reflection on personal environmental values (Whitmarsh and O’Neill, 2010). Cultivating green HABs can be supported through incentives for repeated sustainable actions, such as tiered discounts for consecutive green purchases, to encourage the development of automatic behavioral patterns over time (Yang et al., 2025). Enhancing EP may be achieved through localized impact data, such as neighborhood-level reports on reductions in plastic waste, and visualization tools like QR codes that link products to real-time metrics on emission reductions, thereby making abstract environmental benefits tangible and actionable (Suhartanto et al., 2021). Collectively, these strategies leverage the study’s insights into cognitive, normative, and habitual mechanisms, ensuring that interventions are grounded in the psychological foundations of sustained green consumption.

## 8 Limitations, and future research directions

While this study offers pivotal perspectives on the determinants of consumers’ continuance behavior intention to purchase green products, there are several limitations and future research that should be acknowledged.

First, the cross-sectional nature of this study restricts the capacity to deduce causality among the variables. While the findings indicate substantial correlations, subsequent studies ought to incorporate longitudinal methodologies to more accurately trace the evolution of factors impacting CBI and to assess how these factors ultimately shape real consumer actions over an extended period.

Second, the data for this study were collected through self-reports, which are subject to social desirability biases and recall biases. Consumers may overstate their intentions to engage in sustainable behaviors, leading to potential discrepancies between reported ATTs and actual behaviors. Future research could complement self-reported data with behavioral data, such as actual purchasing behavior or longitudinal tracking of consumer HABs, to enhance the validity of the findings.

Third, the sample used in this study consists of 588 respondents, and while the survey participants were categorized based on their PESI, the study may not fully capture the diversity of consumers’ ATTs and behaviors in different cultural, social, and geographical contexts. The findings may, therefore, be limited in their generalizability to other populations. Future research could expand the sample to include more diverse groups or examine the moderating effects of cultural and socio-economic factors on pro-environmental behavior.

Last, this study specifically focuses on the purchase of green products. While the factors identified in this study are likely applicable to other sustainable behaviors, the findings may not be directly transferable to other environmental behaviors, such as energy conservation, recycling, or transportation choices. Future studies could broaden the scope to include a wider range of sustainable behaviors to gain a more comprehensive understanding of the factors that drive long-term environmental engagement.

## Data Availability

The raw data supporting the conclusions of this article will be made available by the authors, without undue reservation.
