# Supplementary material for: Beyond the first purchase: unpacking the continuance intentions behind sustainable consumption
Source: Front Psychol. 2025 Oct 10;16:1627728. doi: 10.3389/fpsyg.2025.1627728 (PMC12549699; doi:10.3389/fpsyg.2025.1627728)
Supplement: Supplementary file 1 [file Data_Sheet_1.docx]

| **Appendix A.** Measurement items | | |
| --- | --- | --- |
| **Construct** | **Items** | **Reference** |
| Attitude (ATT) | 1. I like the idea of purchasing green products. | Paul et al. (2016) |
|  | 2. Purchasing green product is a good idea. |  |
|  | 3. I have a favourable attitude toward purchasing green version of a product. |  |
| Subjective norms (SN) | 1. Most people who are important to me think I should purchase green products when going for purchasing. |  |
|  | 1. Most people who are important to me would want me to purchase green products when going for purchasing. |  |
|  | 1. People whose opinions I value would prefer that I purchase green products. |  |
|  | 1. My friend’s positive opinion influences me to purchase green product. |  |
| Perceived behavioral control (PBC) | 1. I believe I have the ability to purchase green products. |  |
|  | 2. If it were entirely up to me, I am confident that I will purchase green products. |  |
|  | 3. I see myself as capable of purchasing green products in future. |  |
|  | 4. I have resources, time and willingness to purchase green products. |  |
|  | 5. Green products are generally available in the shops where I usually do my shopping. |  |
|  | 6. There are likely to be plenty of opportunities for me to purchase green products. |  |
| Pre-adoption experience (PAE) | 1. Regarding the last purchase, I bought it because I considered the price charged in each fair offer. | Costa et al. (2021) |
|  | 2. In relation to the last purchase, the percentage discount was the decisive factor for the purchase. |  |
|  | 3. Regarding the last purchase, when I viewed the offer, I paid more attention to the discount percentage and not to the environmental characteristics of the product. |  |
| Environmental perception (EP) | 1. The balance in nature is very delicate and can be easily disturbed. | Mónus (2021) |
|  | 1. When human beings interfere with nature, it often has disastrous consequences. |  |
|  | 3. Human beings must live in harmony with nature so that they can survive better. |  |
|  | 4. Humanity is seriously abusing the environment. |  |
| Habits (HAB) | 1. Shopping at the store where selling green products is something I do frequently. | Hsu et al. (2015) |
|  | 1. Shopping at the store where selling green products is nature to me. |  |
|  | 1. Shopping at the store where selling green products is something I do without thinking. |  |
| Continuance behavior intention (CBI) | 1. I have the intention to repurchase the green products in the future. | Suhartanto et al. (2021) |
|  | 2. I have the intention to repurchase the green products no matter the price. |  |
|  | 3. I have the intention to regularly use the green products in the future. |  |
